# Feeding tea polysaccharides affects lipid metabolism, antioxidant capacity and immunity of common carp (*Cyprinus carpio* L.)

**DOI:** 10.3389/fimmu.2022.1074198

**Published:** 2022-11-24

**Authors:** Guokun Yang, Xiaomin Liang, Jihong Hu, Chengquan Li, Wenpan Hu, Keke Li, Xulu Chang, Yanmin Zhang, Xindang Zhang, Yawei Shen, Xiaolin Meng

**Affiliations:** ^1^ College of Fisheries, Henan Normal University, Xinxiang, China; ^2^ College of Fisheries, Engineering Technology Research Center of Henan Province for Aquatic Animal Cultivation, Henan Normal University, Xinxiang, China; ^3^ Henan JinBaiHe Biotechnology Co., Ltd, Anyang, China

**Keywords:** tea polysaccharide, metabolism, antioxidant, immunity, common carp

## Abstract

Tea polysaccharides plays a role in lipid metabolism, antioxidant capacity and immunity of mammals. To investigate the functions of tea polysaccharides on fish, the common carp (*Cyprinus carpio* L.) was selected as the animal model in this study. In our study, the common carp (45±0.71g) were randomly divided into four groups and were fed fodder with 50% carbohydrate. The common carp were orally administrated with 0 mg/kg BW (control group), 200 mg/kg BW (low-dose group), 400 mg/kg BW (medium-dose group) and 800 mg/kg BW (high-dose group) tea polysaccharide for two week. At the end of experiment, the serum glucose, TG, MDA contents and antioxidase activities were measured by commercial kits. The serum immune factors levels were tested by ELISA. The genes expression levels related to antioxidant capacity, metabolism and immunity were measured by real-time PCR. The results showed that the glucose, TG and MDA contents in serum were significantly decreased by tea polysaccharides treatment. The serum activities of SOD were significantly increased by low-dose tea polysaccharides treatment. The serum activities of GPX were significantly increased by medium-dose tea polysaccharides treatment. The serum levels of IL-1β and TNFα were significantly decreased in the tea polysaccharides treatment group. In the high-dose treatment group, the serum level of TGFβ was significantly increased, and the serum level of IL-12 was markedly decreased. In the hepatopancreas, the expression of acc1, fas, srebp1c, lpl, gys and pparγ were significantly reduced, and the expression of pygl, cat, mnsod, ho-1 and gr were significantly up-regulated in the tea polysaccharides group. In the intestine, the expression of zo-1, occ and gip was significantly up-regulated in the high-dose treatment group. Moreover, the expression of glut2 and sglt1 were significantly down regulated. In the spleen, the expression of il-12, tnfα and il-6 were significantly decreased, and the expression of il-10 and tgfβ was significantly increased by the tea polysaccharides. In the spleen cells, the tea polysaccharides could relieve the LPS-induced immune damage. In conclusion, tea polysaccharides can improve antioxidant capacity, lipid metabolism and immunity of common carp.

## Introduction

Aquaculture provides an essential source of edible protein for humans. With the rapid development of aquaculture, the demand of fishmeal is increasing, which induces the escalating price of fishmeal (1). The study showed that dietary carbohydrate and lipid inclusion at optimal levels could promote protein utilization, prevent lipid oxidation, and save fishmeal protein in the aquatic animal feed (2). However, overloaded dietary carbohydrate induced low feed intake, poor growth performance (3), metabolism dysfunction, impairment of antioxidant capacity and sub-health status of fish (4, 5). Moreover, overloaded dietary carbohydrate will reduce the immunity, and increase the infection with disease of cultured fish (6). In addition, with the expansion of farming scale and environmental degradation, diseases in farming occur frequently, which causes a number of deaths of cultured fish (7). Although the antibiotic can relieve the morbidity of fish, antibiotic resistance has become a severe problem worldwide (8). The frequent occurrence of antibiotic-resistant bacteria in the aquaculture sites is due to the abuse of antibiotics in aquaculture (9). Therefore, the antibiotics were prohibited in aquaculture by the Chinese government because of the various negative effects after July 2020. For these reasons, it is an urgent problem that the new antibiotic substitute has been exploited for use in aquaculture. As the environmental friendly substance, plant extracts are low toxicity, safety, and minimal environmental impacts (10). In aquatic animals, plant extracts play important role in enhancing the immune function, and promote antibacterial, antiviral, antiparasitic activities of the immune system (10). In addition, plant extracts have been as immunostimulant to prevent diseases of aquatic animals in recent years (11).

As the important economic agricultural product, tea possesses multiple beneficial effects, including antioxidant capacity, reduction of cholesterol, protect against cardiovascular disease, anti-microbial, and anti-cancer (12–14). The beneficial effects of tea attribute to its variety of bioactive compounds, including polysaccharides, polyphenols, alkaloids, volatile oils, amino acids, etc (14–18). The tea polysaccharides attracted attention for its bioactivities, such as antioxidant, anti-cancer, anti-radiation, hypoglycemic activities and anti-HIV (14, 15, 19, 20). Tea polysaccharides were mostly heteropolysaccharides, in which a protein *via* N- or O- covalently linkages carries one or more carbohydrate chains attached to a polypeptide backbone (14, 15). The bioactivity study indicated that the tea polysaccharides suppressed the formation and accumulation of fat, and promoted its decomposition to prevent obesity of rats (*Rattus norvegicus*) (21). For example, a report of polysaccharides from green tea of *Huangshan Maofeng* (HMTP) showed that HMTP could protect against liver injury by CCl_4_-induced, and inhibit lipid peroxidation and the increase antioxidant activity in mice (*Mus musculus*) (22). Furthermore, the tea polysaccharides could significantly reduce blood glucose levels, and increase the capacity of glucose tolerance in mice (23, 24).

Common carp is one of the most widely cultivated freshwater fish species all over the world, which is the fourth most cultured fish species in aquaculture (25). The production of common carp accounts for 7.7% of the total fish production in 2018 in the world (26). In addition, the production of common carp reaches 4,411,900 metric tons in 2019 (25). To meet the needs of human for fish, the intensive high-density and high nutrition farming model was rapidly developed. With the farming model and environmental degradation, diseases in fish farming occur frequently, which causes huge losses to the aquaculture industry. As a safe and environmental friendly plant extracts, tea polysaccharides have antioxidant capacity, anti-microbial actions and increases immunity in mammals. But the beneficial effects of tea polysaccharides in fish have never been reported. To assess the biological functions of tea polysaccharides on fish, the common carp was employed as a model in this study. The effects of tea polysaccharides on the immunity, metabolism and antioxidant capacity were evaluated in common carp in our study.

## Materials and methods

### Materials and chemicals

Tea polysaccharides was produced in meilunbio (Dalian, China). Glucose test kit was purchased from Rsbio (Shanghai, China). Triglyceride (TG) test kit was purchased from Dongou (Zhejiang, China). Superoxide dismutase (SOD), glutathione oxidase (GPX), total antioxidant capacity (T-AOC) and methane dicarboxylic aldehyde (MDA) test kits were obtained from Nanjing Jiancheng Bioengineering Institute (Nanjing, China).

### Animals experiment

The experiment process was referred to the previous studies (27, 28). The common carp with an initial body weight (45 ± 0.71g) were obtained from Yanjin Fishery (Yanjin, Henan). Approximately 120 healthy individuals were randomly divided into four groups (30 fish in each group). Before the experiment, fish were acclimated to indoor tanks (diameter: 52 cm, water high: 62cm) at room temperature with recirculating water under a cyclical light-dark photoperiod (12 h: 12 h) for two weeks. Then, the fish were fed with a high carbohydrate diet or the same diet with 200 mg/kg BW/day (low-dose group), 400 mg/kg BW/day (medium-dose group) and 800 mg/kg BW/day (high-dose group) tea polysaccharides by gavage for two weeks. The formulations and compositions of each diet were listed in **Table 1**. All ingredients were weighted individually before mixed thoroughly in a feed drum mixer for 30 min. Then dissolved water was added and mixed to form a loosely shaped dough. The mixture was transformed into pellets using a single screw extruder (Fishery Machinery and Instrument Research Institute, China Academy of Fishery Science, Shanghai, China); the pellets were then air-dried at room temperature and stored at − 20°C.

**Table 1:** Ingredients and proximate composition (% dry matter) of experimental diet.

Ingredients	Percent of total
Casein	33
Fish meal	10
Fish oil	2.5
soybean oil	1.5
Mineral premix^a^	1
Vitamin premix^b^	1
Sodium carboxymethylcellulose	0.5
Glucose	50
Choline chloride	0.5
Total	100
Proximate composition	
Crude protein (%)	34.75
Crude lipid (%)	4.28
Ash (%)	2.46

^a^Vitamin premix (mg or IU/kg diet): vitamin A, 6000 IU; vitamin D3, 2000 IU; vitamin E, 50 mg; ascorbic acid, 200 mg; pantothenic acid, 35 mg; nicotinic acid, 30 mg; thiamine, 15 mg; riboflavin, 15 mg; pyridoxine Hcl, 6 mg; cyanocobalamin, 0.03 mg; menadione, 5 mg; inositol, 200 mg; folic acid, 3 mg; biotin, 0.2 mg.

^b^Mineral premix (mg or g/kg diet): magnesium, 100 mg; iron, 150 mg; zinc, 80 mg; manganese, 20 mg; copper, 4 mg; iodine, 0.4 mg; cobalt, 0.1 mg; selenium, 0.1 mg.

### Biochemical analysis

At the end of the experiment, all fish were anesthetized by MS222 (Sigma, USA), and the blood samples were collected from the caudal vein. After still standing at 4°C at least 30 min, the serum was isolated by centrifugation at 7500 g for 10 min. The serum was stored at −80°C for the detection of immune factors and biochemical analysis. And then, the fish were decapitated. The hepatopancreas, foregut and spleen samples were immediately collected, and snap-frozen in liquid nitrogen and stored at −80°C until RNA extraction. Parts of the hepatopancreas tissue was removed for glycogen contents measure *via* the commercial kit (Jiancheng, China). All animal experiments were approved by the Animal Care Committee of Henan Normal University.

### Serum samples analysis

In the serum, the content of glucose, TG and MDA of all groups was determined by commercial kits (Jiancheng, China). The enzyme activities of SOD, GPX and T-AOC were determined by commercial kits (Jiancheng, China). The experiments were performed according to the manufacturer’s protocol. The levels of IL-1β, IL-6, IL-10, IL-12, tumor necrosis factor α (TNFα) and transforming growth factor β (TGFβ) were measured by ELISA assay referred to pervious study (28).

### Common carp spleen cells isolation and treatment

The common carp spleen cells were isolated by collagenase IV/DNase II digestion method. The experimental method of isolation was referred to previous study (29). The isolated spleen cells were cultured in the 24 wells plate with 1 mL DMEM/F12 medium contained 10% fetal bovine serum (FBS) with the density of 1×10^6^ cells/well. After overnight cultured, the cell medium was replaced to fresh DMEM/F12 without FBS. Before treatment, the cells were cultured for 1 h in the DMEM/F12 without FBS. Then, the cells were treated with LPS (25 μg/mL), tea polysaccharides (400 μg/mL), LPS (25 μg/mL) + tea polysaccharides (400 μg/mL) for 12 h. By the end of the study, the cells were lysed by RNAiso Plus for RNA extraction.

### RNA extract, cDNA synthesis and real-time PCR

The total RNA of the hepatopancreas, gut and spleen were extracted by RNAiso Plus (Takara, Janpa). The total RNA concentration was measured by UV spectrophotometer (Nanodrop 2000, Thermo). 1 μg of total RNA was digested with gDNA Eraser at 42°C for 2 min to eliminate genomic DNA. Then, the first-strand cDNA was synthesized using PrimeScript RT reagent kit (PrimeScript RT reagent kit with gDNA Eraser, Takara). The synthesized first-strand cDNA was used as template for real-time PCR and the primers were shown in **Table 2**.

**Table 2:** Primers used in this study.

Gene	Accession no.	Forward (5’→3’)	Reverse (5’→3’)
*occ1*	KF975606	ATGTTGTCCTTCCCGTGATAAG	TCCGTAAGAACCTCCGTAAGA
*zo-1*	KY290394	AGGAAGTTCTCCCTCGTACTC	CCTCTGTTGTGGTTGAGTGTAG
*sglt-1*	JN867793.1	CTAAAGAAGAGGAGGCAGAGTTG	ACAGACGGTGAGGAGGATAATA
*mnsod*	XM_019111527.1	CGCACTTCAACCCTCAT	CATTGCCTCCTTTACCC
*cat*	JF411604.1	TTCCTGTGGGACGCCTTGT	TCCGAGCCGATGCCTATGT
*gr*	XM_019102099.1	TGGCTGGTATCCTTTCC	TGTCGTCAGGGTCTTTT
*tnfα*	XM_019088899.1	AGCCAGGTGTCTTTCCACAT	ATGTAGCCGCCATAGGAATCG
*IL-6*	XM_019073058.1	CATCTGGGGACGAGGTTCAG	AGGGTTTGAGGAGAGGGGTT
*il-1β*	AB010701.1	CAAACTGGAGCTGTCTTCGC	CTTCACCAGACGCTCTTCGAT
*il-10*	JX524550.1	TTGCTCATTTGTGGAGGGCT	TGTTGCACGTTTTCGTCCAG
*il-12*	AJ480354	TGCTTCTCTGTCTCTGTGATGGA	CACAGCTGCAGTCGTTCTTGA
*tgfβ*	AF136947.1	TGCCTGTTGGGATTTGTGC	AGCCGCCTGCTCTTCATTT
*acc1*	XM_019096370.1	TTCACTGGCGTATGAGGATATC	TCCACCTGTATGGTTCTTTGG
*fas*	GQ466045.1	GACAGGCCGCTATTGCTATT	TGCCGTAAGCTGAGGAAATC
*srebp1c*	KY763985	GACGCCGCTGAACAACTT	TCCTCGGGCTTCTCCACA
*glut2*	XM_019072653.1	GAGGGTCTTTGTGGGAACTATG	GTTTCAGGTACACGCAAGTAGA
*pygl*	XM_019125106.1	TGGTTGACGACGATGCTTTC	ACTGCGCAAACTTCAGCTTG
*gip*	GFWU01032553.1	AGTTTAGCCGCCGTTAC	TTTCTTCTCCCTCTGATTG
*gys*	XM_019090903.1	TTTTGGCCGCTGGTTGATTG	ATAGGGTAGTCCAATGCTGCAC
*lpl*	FJ716101.1	CGCTCCATTCACCTGTTCAT	GCTGAGACACATGCCCTTATT
*pparγ*	XM_019096045.1	TGCAAGGGATTCTTCCGCAG	AACGAATGGCGTTGTGTGAC
*ho-1*	JX257180.1	TCAGCCCATCTACTTCCCTCA	GGCAGGCACTGTTACTCTCT
*18s*	FJ710827.1	GAGACTCCGGCTTGCTAAAT	CAGACCTGTTATTGCTCCATCT
*β-actin*	M25013	CGTGACATCAAGGAGAAG	GAGTTGAAGGTGGTCTCAT

The genes expression levels were evaluated by real-time PCR. Real-time PCR was performed using SYBR green qPCR mix (Bimake, China) on the LightCycler 480 II Sequence Detection System (Roche, Switzerland) according to the manufacturer’s instructions. The real-time PCR reaction was in a total volume of 10 μl and the following conditions were used: 95°C for 5 min; and 40 cycles of 95°C for 15 s, 56°C for 15 s, and 72°C for 30 s. *18S* rRNA are used as the internal reference, and remained stable in various treatments throughout the study. The genes relative expression levels were normalized to *18S* rRNA. The results were calculated by the comparative Ct method (30).

### Statistical analysis

All data are shown as mean ± standard error of the mean (S.E.M). Statistical analysis was performed with SPSS version 18.0 (SPSS Inc., Chicago, IL, USA). One-way ANOVA followed by Fisher’s Least Significance Difference (LSD) test was used to identify the significant difference. A probability value of *P* < 0.05 was considered significant.

## Results

### Serum content of glucose and triglyceride, the activity of antioxidant enzyme and glycogen content in hepatopancreas

**Figure 1:**
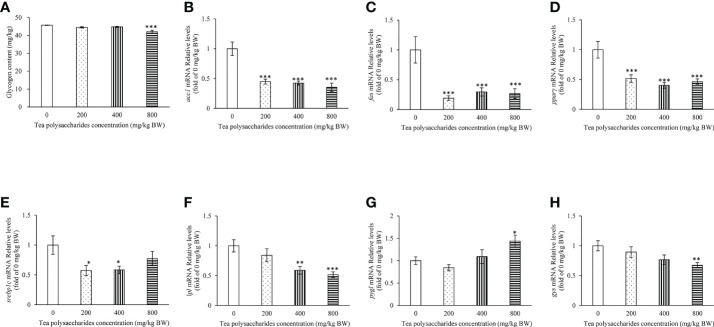
Effects of tea polysaccharides on hepatopancreas glycogen content and genes expression related to lipid metabolism in hepatopancreas. **(A)** Hepatopancreas glycogen content. At end of the experiment, fish were killed and the hepatopancreas was collected for glycogen content detected. **(B–H)** genes expression related to lipid metabolism in hepatopancreas. **(B)** *acc1*; **(C)** *fas*; **(D)** *pparγ*; **(E)** *srebp1c*; **(F)** *lpl*; **(G)** *pygl*; **(H)** *gys*. At end of the experiment, fish were killed and the hepatopancreas was collected for RNA extraction and real-time PCR. All data are shown as mean ± S.E.M. (n = 10-12). Significant differences were indicated by asterisks, *,*P* < 0.05; **,*P* < 0.01; ***,*P* < 0.001.

In our present study, the result showed that the content of glucose and TG in serum was significantly decreased in the tea polysaccharides treatment groups compared to that of control group (**Table 3**). Moreover, the activity of SOD enzyme significantly increase by low-dose tea polysaccharides treatment (**Table 3**). The activity of GPX enzyme significantly increase by medium-dose tea polysaccharides treatment (**Table 3**). However, the contents of MDA were dramatically reduced in the tea polysaccharides groups compared to that of control group (**Table 3**). As shown in the **Figure 1A**, the contents of glycogen in hepatopancreas were markedly decreased in the high-dose group compared to that in the control group.

**Table 3:** Effects of tea polysaccharides on the plasma components of common carp.

	0mg/kg BW	200mg/kg BW	400mg/kg BW	800mg/kg BW
Glucose (mM)	2.81 ± 0.22^a^	1.44 ± 0.20^c^	1.96 ± 0.16^bc^	2.15 ± 0.27^b^
TG (mM)	3.13 ± 0.33^a^	2.28 ± 0.18^b^	2.10 ± 0.18^b^	2.31 ± 0.29^b^
SOD (U/mL)	57.52 ± 3.02^b^	110.56 ± 5.37^a^	80.53 ± 13.86^b^	65.19 ± 9.26^b^
T-AOC (U/mL)	4.07 ± 0.42	4.90 ± 0.44	4.17 ± 0.38	4.75 ± 0.45
MDA (nM)	10.72 ± 1.27^a^	7.42 ± 0.66^b^	7.61 ± 0.44^b^	7.47 ± 0.34^b^
GPX (U/mL)	342.74 ± 37.47^b^	382.64 ± 34.15^ab^	431.60 ± 18.98^a^	324.34 ± 24.84^b^

All data are shown as mean ± S.E.M. (n = 7-8). Significant differences (P<0.05) were indicated by different letters.

### The contents of immune factors in serum

In **Table 4**, the results showed that the contents of IL-1β and TNFα in serum were significantly reduced in the tea polysaccharides treatment groups compared to that of the control group. The content of IL-12 in serum was markedly decreased in the high-dose tea polysaccharides treatment group. However, the content of TGFβ in serum was dramatically elevated in the high-dose tea polysaccharides treatment group compared to that of the control group.

**Table 4:** Effects of tea polysaccharides on the cytokine in plasma of common carp.

	0mg/kg BW	200mg/kg BW	400mg/kg BW	800mg/kg BW
IL-1β (pg/mL)	1014.4 ± 29.0^a^	865.3 ± 41.8^b^	827.0 ± 60.9^b^	783.8 ± 22.2^b^
IL-6 (pg/mL)	41.3 ± 2.3	41.5 ± 2.4	46.3 ± 2.8	43.5 ± 4.6
IL-10 (pg/mL)	25.6 ± 1.8	26.9 ± 1.2	27.5 ± 1.1	26.9 ± 0.9
IL-12 (pg/mL)	183.7 ± 3.9^a^	174.6 ± 7.6^ab^	169.1 ± 6.8^ab^	156.4 ± 5.1^b^
TGFβ (pg/mL)	43.4 ± 1.9^b^	47.8 ± 1.7^b^	47.8 ± 0.9^b^	54.4 ± 1.1^a^
TNFα (pg/mL)	51.0 ± 2.1^a^	44.3 ± 1.7^b^	43.3 ± 1.8^b^	41.9 ± 1.8^b^

All data are shown as mean ± S.E.M. (n = 7-8). Significant differences (P<0.05) were indicated by different letters.

### Effect of tea polysaccharides on expression of the genes related to metabolism in hepatopancreas

The results showed that the expression of fatty acid synthesis gene *acc1*, *fas* and *pparγ* in hepatopancreas was significantly inhibited in the tea polysaccharides treatment groups compared to that of control group (**Figures 1B, C**). The *srebp1c* expression level was markedly decreased by the low- and medium-dose tea polysaccharides treatment (**Figure 1D**). The *lpl* expression level was markedly decreased by the medium- and high-dose tea polysaccharides treatment (**Figure 1F**). Moreover, the *pygl* expression level was significantly increased in the high-dose tea polysaccharides treatment group (**Figure 1G**). However, the *gys* expression level was significantly decreased in the high-dose tea polysaccharides treatment group (**Figure 1H**).

### Effect of tea polysaccharides on expression of the genes related to glucose intake and intestinal barrier in foregut

**Figure 2:**
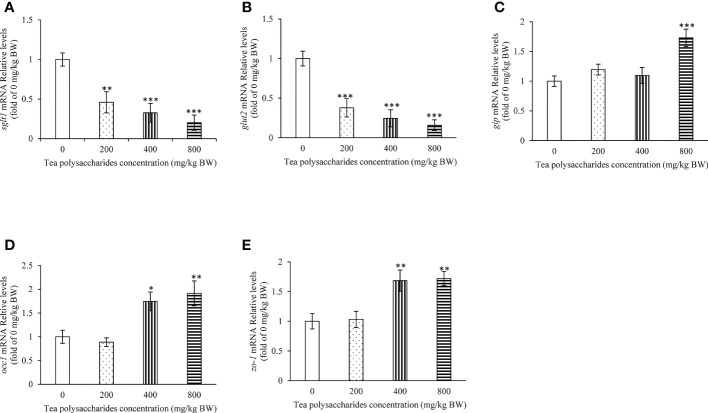
Effects of tea polysaccharides on genes expression related to glucose intake and gut barrier in foregut. **(A)** *sglt1*; **(B)** *glut2*; **(C)** *gip*; **(D)** *occ1*; **(E)** *zo-1*. At end of the experiment, fish were killed and the tissue was collected for RNA extraction and real-time PCR. All data are shown as mean ± S.E.M. (n = 10-12). Significant differences were indicated by asterisks, *,*P* < 0.05; **,*P* < 0.01; ***,*P* < 0.001.

In the foregut, the expression of *sglt1* and *glut2* was dramatically inhibited by the tea polysaccharides treatment (**Figures 2A, B**). Moreover, the *gip* expression level was significantly promoted by the high-dose tea polysaccharides treatment (**Figure 2C**). In the foregut, the mRNA levels of *occ1* and *zo-1* were dramatically increased in the medium- and high-dose tea polysaccharides treatment groups (**Figures 2D, E**).

### Effect of tea polysaccharides on expression of the genes related to antioxidant in hepatopancreas

**Figure 3:**
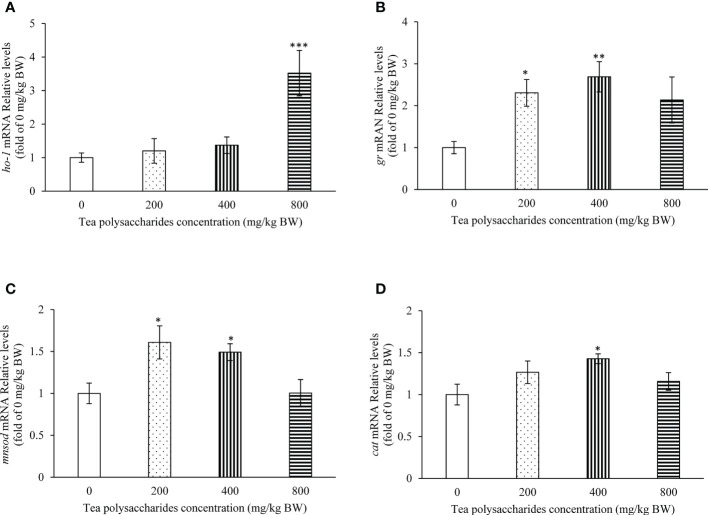
Effects of tea polysaccharides on genes expression related to antioxidant capacity in hepatopancreas. **(A)** *ho-1*; **(B)** *gr*; **(C)** *mnsod*; **(D)** *cat*. At end of the experiment, fish were killed and the hepatopancreas was collected for RNA extraction and real-time PCR. All data are shown as mean ± S.E.M. (n = 10-12). Significant differences were indicated by asterisks, *,*P* < 0.05; **,*P* < 0.01; ***,*P* < 0.001.

In the **Figure 3**, the results showed that the *ho-1* expression level in hepatopancreas was significantly increased in the high-dose tea polysaccharides treatment group compared to that of control group (**Figure 3A**). In the low and medium-dose tea polysaccharides treatment groups, the expression of *gr* and *mnsod* were markedly promoted compared to that of control group (**Figures 3B, C**). In addition, the *cat* expression level was significantly up-regulated in hepatopancreas by the medium-dose tea polysaccharides treatment (**Figure 3D**).

### Effect of tea polysaccharides on expression of the genes related to immunity in spleen

**Figure 4:**
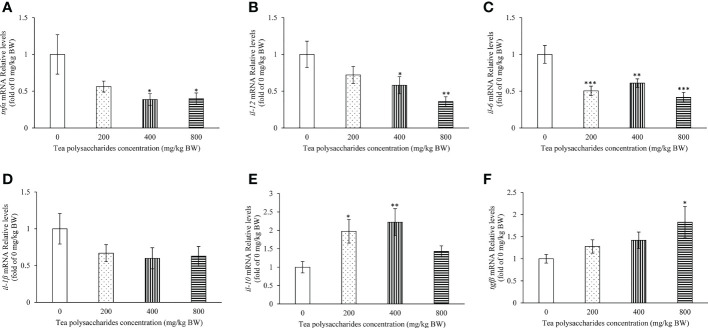
Effects of tea polysaccharides on genes expression related to immunity in spleen. **(A)** *tnfα*; **(B)** *il-12*; **(C)** *il-6*; **(D)** *il-1β*; **(E)** *il-10*; **(F)** *tgfβ*. At end of the experiment, fish were killed and the hepatopancreas was collected for RNA extraction and real-time PCR. All data are shown as mean ± S.E.M. (n = 10-12). Significant differences were indicated by asterisks, *,*P* < 0.05; **,*P* < 0.01; ***,*P* < 0.001.

The effect of tea polysaccharides on expression of immune-related genes was detected in the spleen tissue of common carp. The results showed that the mRNA levels of *tnfα* and *il-12* were significantly inhibited in the medium- and high-dose tea polysaccharides treatment groups (**Figures 4A, B**). The *il-6* expression level was significantly decreased in the tea polysaccharides treatment groups (**Figure 4C**). Moreover, the expression of *il-10* was markedly promoted in the low- and medium-dose tea polysaccharides treatment groups (**Figure 4E**). The expression of anti-inflammatory factor *tgfβ* was significantly increased by the high-dose tea polysaccharides treatment (**Figure 4F**).

### Effect of tea polysaccharides and LPS on expression of the genes related to immunity in common carp spleen cells

**Figure 5:**
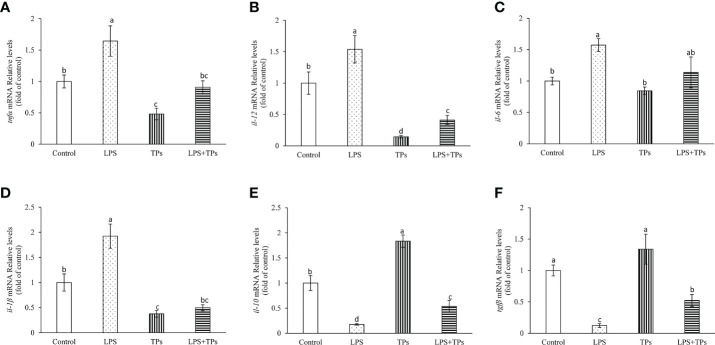
Effects of LPS and tea polysaccharides on genes expression related to immunity in common carp spleen cells. **(A)** *tnfα*; **(B)** *il-12*; **(C)** *il-6*; **(D)** *il-1β*; **(E)** *il-10*; **(F)** *tgfβ*. The cells were seeded in 24-well plates at 1×10^6^ per well in 1mL DMEM/F12 with 10% FBS. The next day, cells were placed in DMEM/F12 without FBS for 1 h. Then, the cells were treated with LPS (25 μg/mL), tea polysaccharides (400 μg/mL), LPS (25 μg/mL) + tea polysaccharides (400 μg/mL) for 12 h. All data are shown as mean ± S.E.M. (n = 5-6). Significant differences (*P*<0.05) were indicated by different letters.

The genes expression of *tnfα*, *il-1β*, *il-6* and *il-12* were significantly increased in common carp spleen cells by treatment with LPS compared to those in control group (**Figures 5A–D**). By tea polysaccharides treatment, the mRNA levels of *tnfα*, *il-1β*, *il-6* and *il-12* were inhibited in spleen cells. Moreover, the promoted mRNA levels of *tnfα*, *il-1β*, *il-6* and *il-12* in spleen cells were alleviated in the LPS and tea polysaccharides group (**Figures 5A–D**). The *il-10* and *tgfβ* expression were markedly decreased in common carp spleen cells by treatment with LPS (**Figures 5E, F**). The *il-10* expression was significantly increased by treatment with tea polysaccharides (**Figure 5E**). Furthermore, the inhibited mRNA levels of *il-10* and *tgfβ* were alleviated in the LPS and tea polysaccharides treatment group (**Figures 5E, F**).

## Discussion

As a group of heteropolysaccharides extracted from tea, tea polysaccharides reveals multiple beneficial bioactivity in previous studies (14, 15). In our study, the serum glucose levels were significantly decreased in the tea polysaccharides treatment groups. The result was similar to that in previous studies. In mice, the blood glucose level was significantly decreased after injection with tea polysaccharides (31). Furthermore, the blood glucose content of alloxan-induced diabetic mice was markedly reduced after four-week oral administration of puerh tea polysaccharides (PTPS) (32), and was suppressed increase after six days oral administration of green tea polysaccharides (GTPS) (33). In addition, the serum glucose levels were decreased by daily oral administration of tea polysaccharides in diabetic and non-diabetic mice (23, 34, 35). These results indicate tea polysaccharides can reduce blood glucose level in mammals and fish. Previous study suggested that the reduction in intestinal glucose transport by tea polysaccharides was mainly mediated by the biochemical inhibition of transport activity (36). In our study, the results also showed that the tea polysaccharides can decrease the *sglt1* and *gult2* genes expression in the foregut. We speculate that tea polysaccharides reduce the serum glucose by lowing the glucose transport in gut of common carp.

The antioxidant activity of tea polysaccharides was reported in previous studies (14, 15). In our study, the enzyme activities of serum SOD and GPX were promoted in the tea polysaccharides groups. However, the contents of MDA were significantly reduced in the tea polysaccharides groups. A report showed that the serum level of T-SOD was significantly promoted in rats, and the elevated serum content of MDA was attenuated in the green tea polysaccharides treated group (37). In addition, pretreatment with Keemun black tea polysaccharides (KBTP), the hepatic T-SOD and GSH levels were reduced, and the MDA content was decreased in CCl_4_-intoxicated mice (38). Moreover, the study showed that the contents of MDA were reduced, and the SOD, catalase and GPX activities were increased in the plasma, liver and heart of mice after treatment with crude tea polysaccharides for 30 days (39). Furthermore, compared to MC (HFD without additional treatment) group, the content of MDA was significantly decreased and the GPX and CAT activities were increased in the Chinese Liupao tea polysaccharides (CLTPS) treatment groups (40). It is indicated that the antioxidant activity of tea polysaccharides can implement in the carbohydrate-, lipid-, toxicant-induced or normal physiological status. Furthermore, tea polysaccharides can increase the genes expression related to antioxidant capacity (*ho-1*, *gr*, *cat* and *mnsod*) in the liver of common carp in our study. Based on the above results, we speculate that tea polysaccharides increase the antioxidant capacity by lowing the levels of MDA and increasing activities and gene expression of antioxidase of common carp.

It is a crucial activity that tea polysaccharides promote immunity (14, 15, 36, 41). Tea polysaccharides activate immune cells to secrete various biological reaction mediators, such as cytokines, free radicals, and lyases (36). In the present study, the contents of IL-1β, TNFα and IL-12 were significantly decreased, and the level of TGFβ was markedly promoted in the serum of common carp after tea polysaccharides treatment. Furthermore, the contents of IL-2, IL-6, IL-10 and IFN-γ were significantly reduced in the colitis-associated cancer (CAC) mice by treatment with tea polysaccharides, and the levels of IL-10 were markedly increased in tea polysaccharides groups compared to that in Azoxymethane/Dextran sulfate sodium (AOM/DSS) group (42). Moreover, the serum IL-6 and TNFα levels were decreased, and the serum IL-2, IL-4 and IL-10 levels were increased in gastric cancer mice after tea polysaccharides treatment (43). In addition, the study of Yuan and his colleagues showed that IL-6 and TNFα levels were significantly decreased in mouse splenocytes treated with tea polysaccharides compared with that in native control (NC) group (44). Based on those results, it is indicated that improving the activity of antioxidant enzymes of tea polysaccharides was not only in mammals but also in the fish. In addition, the expression of *il-1β*, *tnfα*, *il-6* and *il-12* were significantly inhibited, and the expression of *il-10* and *tgfβ* were dramatically increased in the spleen of common carp in the tea polysaccharides groups. In the common carp spleen cells, the tea polysaccharides could relieved LPS-induced immune related genes expression. The previous study also showed that the expression levels of *il-6* and *tnfα* were reduced in white adipose tissue of rats in the tea polysaccharides treatment groups (37). Based on the above study, it is manifested that tea polysaccharides plays immunocompetence by regulating the activity of antioxidant enzymes and expression of immune factors.

Tea polysaccharides also have a fat-lowing effect (36). In our study, the serum TG contents were also significantly reduced in the tea polysaccharides groups. The activity of reducing TG levels of tea polysaccharides was reported in previous studies. For instance, the black tea polysaccharides significantly reduced the TG content in serum and liver of rats compared to that in model control (Wu et al., 2016). The HMTP can decrease the CCl_4_-elevated level of serum TG (22), and administration of GTPS or black tea polysaccharides (BTPS) in mice before the CCl_4_ injection can resist the CCl_4_-induced increases in the level of TG (15). In addition, the contents of serum TG were markedly reduced in mice after Ilex Kuding tea polysaccharides (IKTP) treated with 200, 400 and 800 mg/kg BW (45). Those results indicate that the activity of reducing TG of tea polysaccharides is ubiquitous. In addition, the expression of *lpl*, *acc1*, *fas*, *srebp1c* and *pparγ* were significantly decreased in the tea polysaccharides treatment groups. Our results were similar to that in previous study, in which the tea polysaccharides effected the gene expression related to fat metabolic pathways of rats, and suppressed the accumulation and formation of fat and promoted lipolysis to prevent obesity (Wu et al., 2016). Moreover, a study reported that green tea polyphenols prevent HFD-induced obesity by increasing adiponectin levels, and alleviation of PPARγ phosphorylation (46). Another study reported that tea-supplemented reduced body fat mass of rats and down-regulated the expression of *pparγ*, *c/ebpβ* and *lpl* (47). Furthermore, the results revealed that the lipogenic-related genes expression was affected by kuding tea treatment, and that the expression of *pparγ* and lipogenic genes were inhibited in the liver of mice (48). From above results, it is indicate that the fat-lowing activity of tea polysaccharides is exerted by regulating the expression of lipid metabolism related genes in mammals and fish.

In the present study, the expression of *occ* and *zo-1* were significantly increased in the foregut of common carp in the tea polysaccharides groups. The expression of *gip* was increased, and *sglt1* and *glut2* expression were decreased in the foregut in the tea polysaccharides groups. SGLT1 and GLUT2 are the important glucose transporter in the intestines. In a previous study, the intestinal glucose transport was significantly decreased by treatment with green tea extract (GTE), water soluble polysaccharides derived from green tea (WSP), and GTE+WSP. The expression of *sglt1* was markedly decreased in the Caco-2 cells by treatment with wheat starch + GTE + WSP (49). Moreover, the protein expression of PI3Kp85, p-Akt and GLUT4 in diabetic mice were increased in the liver by orally gavage with tea polysaccharides, and the serum glucose level was accompanied decreased (34). In addition, the expression of *gys* was inhibited and the hepatopancreas glycogen content was decreased in the tea polysaccharides treatment group. The expression of *pygl* was significantly increased in the tea polysaccharides treatment group. The decreased hepatopancreas glycogen content was caused by the decreased glycogen synthesis (*gys*) and increased glycogenolysis (*pygl*) in hepatopancreas and decreased glucose absorption (*sglt1* and *glut2*) in foregut. It is indicated that the absorption of glucose and glycogenesis were affected by tea polysaccharides *via* regulating the related-genes expression. Intestinal barrier function is an important aspect for intestinal health. The study of cyclophosphamide (Cy)-induced BALB/c mice showed that the colonic TLR4/MyD88/NF-κB p65 and JAK2/STAT3 pathway was activated by pectic heteropolysaccharides and the expression of *claudin1*, *claudin5* and *occludin1* were significantly increased (50). In addition, the expression levels of *claudin1* and *claudin5* were significantly promoted in the colonic tissues of mice in the polysaccharides from the tea flower (TFPS) treatment group (51). Those results indicated that the tea polysaccharides play beneficial role in intestinal health by regulating intestinal barrier related genes expression. The regulatory mechanism underlying the impact of tea polysaccharides on intestinal barrier related genes expression of fish should be investigated in future studies.

## Conclusion

In conclusion, our present results suggested that tea polysaccharides promoted immunity, antioxidant capacity and intestinal barrier function and reduced lipogenesis and glucose transporter of common carp. The results of this study will provide a theoretical foundation of tea polysaccharides application in aquaculture.

## Data availability statement

The datasets presented in this study can be found in online repositories. The names of the repository/repositories and accession number(s) can be found in the article/supplementary material.

## Ethics statement

The animal study was reviewed and approved by Animal Care and Use Ethics Committee of the Henan Normal University.

## Author contributions

GY, XL, JH: conceived and performed the experiments, formal analysis, writing, and original draft. XM, GY: Reviewing and editing. WH, KL, XC, YZ: Supervision, reviewing, and editing. CL, YS, XZ: analyzed the data. All authors contributed to the article and approved the submitted version.

## Funding

This work was supported by the National Natural Science Foundation of China (U1904118, 32273149), the Natural Science Foundation of Henan Province (212300410174).

## Conflict of interest

Authors WH and KL were employed by Henan JinBaiHe Biotechnology Co., Ltd.

The remaining authors declare that the research was conducted in the absence of any commercial or financial relationships that could be construed as a potential conflict of interest.

## Publisher’s note

All claims expressed in this article are solely those of the authors and do not necessarily represent those of their affiliated organizations, or those of the publisher, the editors and the reviewers. Any product that may be evaluated in this article, or claim that may be made by its manufacturer, is not guaranteed or endorsed by the publisher.

## Glossary

**Table:**

TG	triglyceride
MDA	malondialdehyde
SOD	superoxide dismutase
GPX	glutathione peroxidase
ACC1	acetyl-CoA carboxylase 1
FAS	fatty acid synthase
SREBP1c	sterol regulatory element binding protein 1c
LPL	lipoprotein lipase
PPARγ	peroxisome proliferator activated receptor γ
PYGL	glycogen phosphorylase
CAT	catalase
MnSOD	manganese superoxide dismutase
HO-1	heme oxygenase-1
GR	glutathione reductase
OCC	occludin
GLUT2	glucose transporter 2
GYS	glycogen synthase
SGLT1	sodium/glucose cotransporter 1
HMTP	Huangshan Maofeng
GIP	glucose-dependent insulinotropicploypeptide
PTPS	puerh tea polysaccharides
GTPS	green tea polysaccharides
BTPS	black tea polysaccharides
KBTP	Keemun black tea polysaccharides
CLTPS	Chinese Liupao tea polysaccharides
CAC	colitis-associated cancer
AOM/DSS	Azoxymethane/Dextran sulfate sodium
NC	native control
GTE	green tea extract
WSP	water soluble polysaccharides derived from green tea
TFPS	polysaccharides from tea flower
IKTP	Ilex Kuding tea polysaccharides
TPs	tea polysaccharides.
